# Predicting sepsis using a combination of clinical information and molecular immune markers sampled in the ambulance

**DOI:** 10.1038/s41598-023-42081-6

**Published:** 2023-09-10

**Authors:** Kedeye Tuerxun, Daniel Eklund, Ulrika Wallgren, Katharina Dannenberg, Dirk Repsilber, Robert Kruse, Eva Särndahl, Lisa Kurland

**Affiliations:** 1 School of Medical Sciences, Faculty of Medicine and Health, Örebro University, Örebro, Sweden; 2 Inflammatory Response and Infection Susceptibility Centre, (iRiSC), Faculty of Medicine and Health, Örebro University, Örebro, Sweden; 3 Gustavsbergs Vårdcentral, Gustavsberg, Stockholm, Sweden; 4 Department of Clinical Research Laboratory, Faculty of Medicine and Health, Örebro University, Örebro, Sweden; 5 Department of Emergency Medicine, Örebro University Hospital, Örebro, Sweden

**Keywords:** Immunology, Molecular biology, Diseases, Health care, Medical research, Predictive markers

## Abstract

Sepsis is a time dependent condition. Screening tools based on clinical parameters have been shown to increase the identification of sepsis. The aim of current study was to evaluate the additional predictive value of immunological molecular markers to our previously developed prehospital screening tools. This is a prospective cohort study of 551 adult patients with suspected infection in the ambulance setting of Stockholm, Sweden between 2017 and 2018. Initially, 74 molecules and 15 genes related to inflammation were evaluated in a screening cohort of 46 patients with outcome sepsis and 50 patients with outcome infection no sepsis. Next, 12 selected molecules, as potentially synergistic predictors, were evaluated in combination with our previously developed screening tools based on clinical parameters in a prediction cohort (n = 455). Seven different algorithms with nested cross-validation were used in the machine learning of the prediction models. Model performances were compared using posterior distributions of average area under the receiver operating characteristic (ROC) curve (AUC) and difference in AUCs. Model variable importance was assessed by permutation of variable values, scoring loss of classification as metric and with model-specific weights when applicable. When comparing the screening tools with and without added molecular variables, and their interactions, the molecules per se did not increase the predictive values. Prediction models based on the molecular variables alone showed a performance in terms of AUCs between 0.65 and 0.70. Among the molecular variables, IL-1Ra, IL-17A, CCL19, CX3CL1 and TNF were significantly higher in septic patients compared to the infection non-sepsis group. Combing immunological molecular markers with clinical parameters did not increase the predictive values of the screening tools, most likely due to the high multicollinearity of temperature and some of the markers. A group of sepsis patients was consistently miss-classified in our prediction models, due to milder symptoms as well as lower expression levels of the investigated immune mediators. This indicates a need of stratifying septic patients with a priori knowledge of certain clinical and molecular parameters in order to improve prediction for early sepsis diagnosis.

**Trial registration**: NCT03249597. Registered 15 August 2017.

## Introduction

Sepsis is defined as a life-threatening organ dysfunction due to a dysregulated host response to infection^1^. Despite advances in medical care, the mortality of sepsis ranges from 10 to 40%^1–3^. In Sweden, sepsis affects approximately 70,000–80,000 people annually^4,5^, while the corresponding number globally is almost 50 million^6^. For this reason, WHO has called for a global action on sepsis^7^ and early diagnosis is one crucial aspect to consider for improved care of the septic patient.

Timely treatment is shown to reduce mortality and improve outcomes in patients with sepsis and septic shock^8–10^; early treatment requires early identification. Since more than half the patients with severe sepsis are transported to hospital by ambulance^11,12^, identification during this first physical contact with health care should improve patient outcome. This is supported by studies demonstrating that the time to treatment is reduced when the septic patient is identified in the prehospital setting^11,13^.

Identification of sepsis in the prehospital setting is currently based on clinical judgment, which is proven inadequate^14^. Identification can be increased when using screening tools, however, to date, there are few screening tools available^15,16,17^, and few have been developed for use in the ambulance. We have previously, in the prospective study Predict Sepsis, developed a set of three Predict Sepsis screening tools based on symptoms and/or vital signs in the prehospital setting^18^. However, as one third of the patients with severe infection exhibit normal vital signs, screening tools based mainly on vital signs present a problem^19^. Furthermore, parameters reflecting the underlying pathophysiology are not included in this type of screening tools.

Immune dysregulation in sepsis is currently a field of intense research including both excessive inflammation and immunosuppressive reactions to the underlying infection^20^. A large number of markers for diagnostic and prognostic purposes have been studied, including immune, vascular, organ, coagulation, and cellular markers but few have been found to increase sepsis identification^21,22^. One likely reason is that these biomarkers have typically been studied as single markers in isolation, i.e., not taking complex pathophysiological interactions into consideration^23,24^. In the current study, the aim was to evaluate the additional predictive values of immunological molecular markers to our previously developed Predict Sepsis screening tools.

## Materials and methods

### Study design

**Figure 1:**
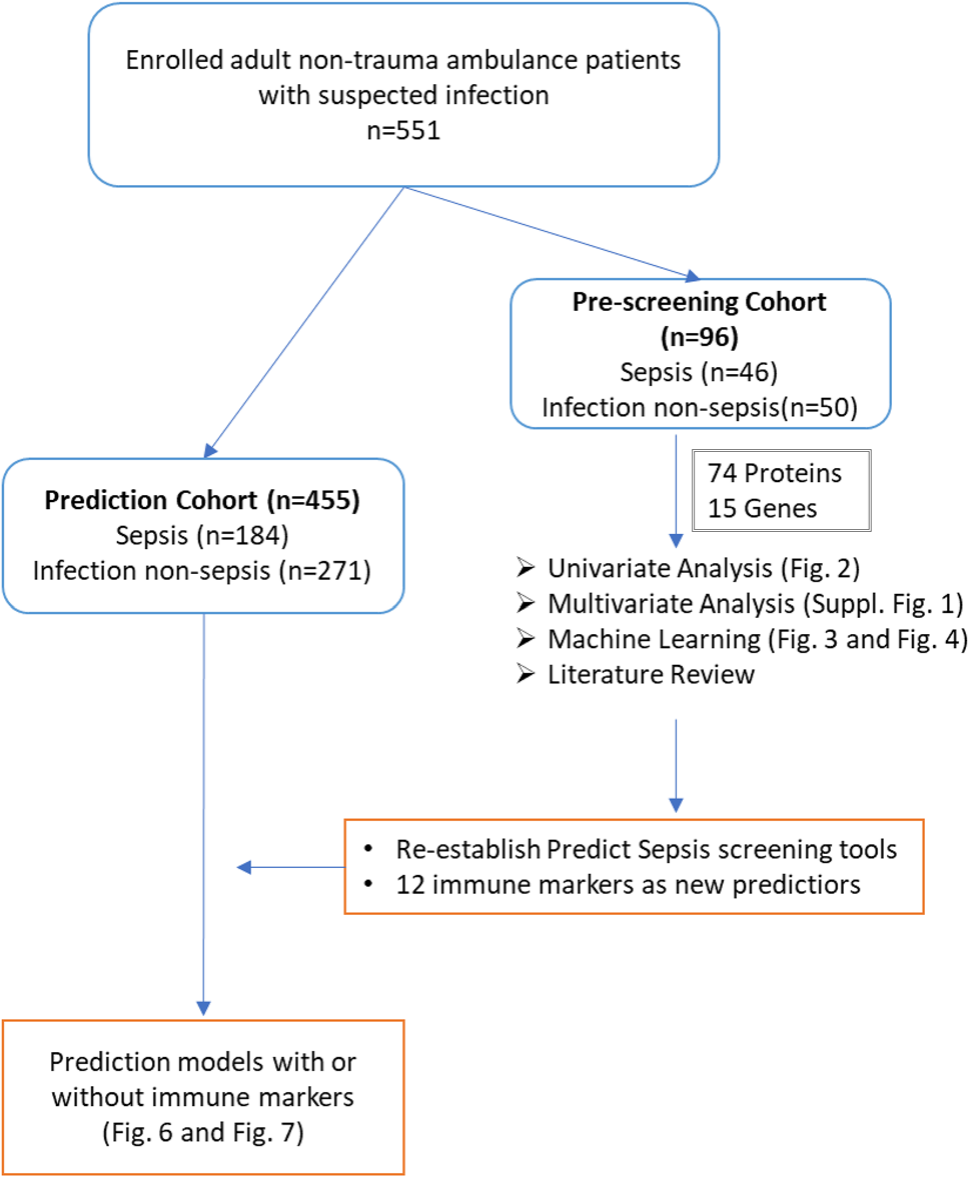
Schematic illustration of the outline of current study.

This current study is part of the Predict Sepsis study, which is a prospective cohort study in the ambulance setting, with patient inclusion between 2017 and 2018, in Stockholm County, Sweden (for details see Wallgren et al.^18^). The study received approval from the Stockholm Regional Ethical Review Board (reference number 2016/2001–31/2 and 2018/2202). Written informed consent was obtained from all participants. The study was registered at ClinicalTrials.gov, identifier: NCT03249597. The outline of the current study is illustrated in Fig. 1.

### Study population

The study included a total of 551 adult, non-trauma patients assessed to have a new onset infection according to clinical judgment made by the ambulance personnel. The inclusion and exclusion criteria have been published elsewhere^18^. A selection of candidate molecular markers reflecting immune responses were performed in a smaller group of consecutively included patients, i.e., the screening cohort (n = 96). The selected candidate molecular markers from the screening cohort were analyzed in the remaining patients (n = 455), i.e., the prediction cohort, and used as predictors in combination with the available clinical variables measured in the ambulance in the final prediction modeling (details of cohorts, see Table 2).

### Blood sampling

Blood was drawn in EDTA-tubes in the ambulance, and at arrival to the hospital bound emergency departments (EDs), tubes were centrifuged, aliquoted, and frozen in − 70 °C in biobank. Furthermore, blood was drawn directly into PAXgene tubes (PreAnalytix, GmbH, Hombrechtikon, Switzerland), with immediate stabilization of intracellular RNA, before being frozen in − 70 °C at arrival to the ED.

### Quantification of circulating inflammatory mediators

Initially, a total of 71 circulating proteins were analyzed within the screening cohort using U-PLEX Biomarker Group 1 kits (Meso Scale Discovery, Rockville, MD) detected by electrochemiluminescence in Meso QuickPlex SQ 120 (Meso Scale Discovery), according to the manufacturer's instructions. Three additional proteins, CXCL6, HGF, and TGF-α were measured by Human Magnetic Luminex Assay (R&D systems, Inc. Minneapolis, MN), according to the manufacturer's instructions. The samples were analyzed on a Luminex^®^200™ instrument (Invitrogen, Merelbeke, Belgium), and the data were collected using the xPONENT 3.1™ software (Luminex Corporation, Austin, TX). Later in the prediction cohort, nine selected mediators, i.e., CCL24, CX3CL1, CCL27, CCL11, IL-17AF, IL-17A, IL-1Ra, TNF, and CCL19, were analyzed using customized U-PLEX kits (Meso Scale Discovery, Rockville, MD).

All values were expressed as pg/mL deduced from the standard curve, using a 5-parameter logistic algorithm. Values below the detection limit were given half the value of the detection limit. All samples were run in duplicates and a coefficient of variation (CV) below 20% was considered acceptable. In Supplementary Table 1, the average CVs and detection limits of the nine proteins analyzed in the prediction cohort are listed.

### RNA extraction and cDNA extraction

All samples were arranged in random order prior to RNA extraction. For the screening cohort, RNA extraction was done using PAXgene Blood RNA Kit (Cat. No. 172021754, Qiagen, GmbH, Hilden, Germany), according to the manufacturer's instructions. RNA quality and concentration was measured with NanoDrop 2000 (Thermo Fisher Scientific, MA, USA) and 2100 Bionalyzer (Agilent, CA, USA). The A260/A280 ratios were above 1.7 and the RNA integrity number (RIN) values were above 7. cDNA was synthesized using the High-Capacity cDNA Reverse Transcription Kit (Applied Biosystems, CA, USA) in a LifePro Thermal Cycler (Bioer, Hangzhou, P.R. China), using 200 ng RNA per 20 μL reaction. Gene expression was performed in a Quantstudio 7 Flex Real-Time PCR system (Applied Biosystems, CA, USA), using TaqMan Gene Expression Assays and TaqMan Fast Universal PCR Master Mix (Applied Biosystems, CA, USA) in a 20 μL reaction, according to the manufacturer’s instructions.

For the prediction cohort, the samples together with a negative extraction control (consisting of RNase-free water) were extracted using the QIAsymphony extraction robot (Qiagen GmbH, Hilden, Germany). RNA was eluted in a total volume of 80 μL and immediately denatured at 65 °C for 10 min using a thermal cycler (T-100, BioRad, CA, UAS). Sample concentration and purity were determined by spectrophotometry on the Lunatic instrument (Unchained Labs, CA, USA) and RNA integrity was analyzed on capillary gel electrophoresis, Fragment Analyzer (Agilent, CA, USA) using RNA Standard Sensitivity Fragment Analyzer kit (Cat. No. DNF-471, Agilent, CA, USA). The A260/A280 ratios were above 1.5 except for 21 samples. These samples did not turn out as outliers in neither univariate nor multivariate analyses, thus were not excluded. None of the negative control samples (ENTCs) showed cross-contamination. All samples were reversed transcribed into cDNA using the TATAA GrandScript cDNA Synthesis Kit (Cat. No. A103, TATAA Biocenter AB, Gothenburg, Sweden). The reverse transcription was performed using 450 ng RNA per 20 μL reaction.

### Real-time PCR

In the screening cohort, a total of 15 genes encoding inflammatory mediators, inflammasome components, and transcription factors (*PYCARD, CASP1, NLRP3, IL1B, IL18, TNF, IL6, IL10, IL1RN, HLA-DRA, HIF1A, SPI1, EPAS1, SIRT1, NFKBIA*) were analyzed using qPCR. Gene expression was performed in a Quantstudio 7 Flex Real-Time PCR system (Applied Biosystems, CA, USA), using TaqMan Gene Expression Assays and TaqMan Fast Universal PCR Master Mix (Applied Biosystems, CA, USA), according to the manufacturer’s instructions (TaqMan assay IDs are listed in Supplementary Table 2). *HPRT1* was used as a reference gene, determined by NormFinder R package (MOMA, Aarhus University Hospital, Denmark) for normalization among a total of three candidate reference genes. All samples of the study were analyzed in duplicates, and the mean quantity values were used in further data analysis. The accepted CV of technical sample replicates was ≤ 15%. Samples with a CV > 15% for each specific assay were re-analyzed. Cycle threshold (CT) cut-off value was set to 35 and all reactions had an efficiency between 90 and 110%. In all cases, gene expression levels were obtained from a six-point serially four-fold diluted calibration curve. The calibration curve was developed from cDNA of PBMCs stimulated by 1 μg/mL LPS.

In the prediction cohort, three genes, *EPAS1*, *HIF1A,* and *NLRP3* were analyzed using assays designed and validated by TATAA Biocenter AB. qPCR was performed with TATAA SYBR®GrandMaster Mix Low Rox (Cat. No. TA01, TATAA Biocenter AB, Gothenburg, Sweden) in 10 μL reaction volume. Human ValidPrimeTM (Cat. No. A105P10, TATAA Biocenter AB, Gothenburg, Sweden) was used to monitor and correct for contaminating gDNA^25^. An inter-plate calibrator (Cat. No. IPC250S, TATAA Biocenter, Gothenburg, Sweden) was run on each plate to be able to correct for inter-run differences.

All samples were run in duplicates in 384-well plate format using QuantStudio™ 7 Pro Real-Time PCR system (384-well, ThermoFisher Scientific). The pipetting was performed by a pipetting robot OT-2 (Opentrons, NY, USA). qPCR raw data were pre-processed and analyzed with GenEx software v.7 (MultiD Analyses AB, Gothenburg, Sweden). The limit of quantification of the assays were determined using standard dilution series for which the relative standard deviation of a replicate was < 35%. The accepted standard deviation of technical sample replicates was ≤ 0.5, whereas the accepted standard deviation of the IPC (Inter Plate Calibrator) replicates was ≤ 0.2. Samples with a standard deviation > 0.5 for each specific assay were re-analyzed. Three reference genes, beta-glucuronidase (GUSB), peptidyl-propyl isomerase A, cyclophilinA (PPIA), and ubiquitin C (UBC) were selected from a list of 12 reference gene candidates using the geNorm and NormFinder functions in GenEx software v.7 (MultiD Analyses AB). The relative gene expression was calculated using the delta CT method.

### Statistical modeling and data analysis

In the data analysis pipeline, clinical and molecular variables were assessed with regard to their differences between sepsis and non-sepsis cases and regarding their quality as predictors, using univariate and multi-variable models. For all models, prediction performance was measured in a nested cross-validation approach as AUCs for the hold-out testing set. Finally, variable importance measures, as eligible for the different analysis methods, were applied.

This data analysis pipeline was first run on data from the screening cohort, followed by a consensus variable selection for further analysis in the prediction cohort. Then, the pipeline was run again, this time only involving the selected molecular variables, on the data from the prediction cohort, with prediction performances and variables importance reported as before.

### Selection of molecular variables in the screening cohort

Based on screening cohort data, molecular variables as synergistic predictors with the clinical parameters were selected through a stepwise process of (i) univariate analyses, (ii) multivariate analyses and (iii) literature review. From this process, a weighted curation was performed for the final selection of molecular variables, which were used for further analysis in the prediction cohort.

The univariate variable selection of the most relevant molecular variables was performed by fitting individual mixed effect models (lmerTest package in R; mixed effects model with the sepsis/non-sepsis as fixed effect and sex as random effect) of the 74 inflammatory mediators as well as the expression levels of 15 genes, to differentiate between non-septic and septic patients, followed by the false discovery rate (FDR) estimation for multiple comparisons. Molecular variables with fold-changes (FC) above the thresholds, set to FC ˃ 1.2 for proteins and FC > 2.0 for mRNAs, and a Benjamini–Hochberg FDR ˂ 0.05, were selected as candidates for analysis in the prediction cohort.

The multivariate variable selection of the most relevant molecular variables was performed by machine learning implemented with a nested cross-validation workflow assessing variables as classifiers of non-septic and septic patients. A set of seven different machine learning algorithms were trained in parallel and tested to evaluate different algorithms with regards to classification performance on 7 different variable sets. The variable sets were; (a) all molecular variables, (b–d) previously reported Predict Sepsis screening tools 1, 2 and 3 (using un-categorized original values of the clinical parameters presented by previous study^18^, summary of parameters in the screening tools see Table 1), and (e–g) combining Predict Sepsis screening tools 1, 2 and 3 with the molecular variables. In addition, two-way interaction between all variables were created by multiplying variables for evaluation of interaction effects.

**Table 1:** Parameters of the predict sepsis screening tools^18^.

Predict sepsis screening tool 1	Predict sepsis screening tool 2	Predict sepsis screening tool 3
Acute altered mental status	Acute altered mental status and/or GCS	RR
Gastrointestinal symptoms	Gastrointestinal symptoms	SpO2
GCS	SBP	HR
SBP	Temp	GCS
Temp		SBP
Lactate		Temp

*SBP* systolic blood pressure, *GCS* Glasgow coma scale, *Temp* temperature, *HR* heart rate, *RR* respiratory rate.

Briefly, all variables included had missing values below 20%. Data were partitioned into training (75%) and testing (25%) sets. Each set of data was standardized, and knn-imputation of missing values and class balanced with Synthetic minority over-sampling technique (SMOTE; themis package in R) was performed on the training set. Thereafter, penalized regularized logistic Lasso regressions (Lasso; glmnet package in R), Random forests (ranger package in R), XGBoosted trees (xgboost package in R), Neural network (nnet package in R), Naïve Bayes (klaR package in R) and lightGBM (bonsai package in R), were in parallel trained with hyperparameter tuning. Hyperparameters for Lasso (penalty); Random Forest (number of variables included in each random tree and minimum n for split); XGBoosted trees (number of variables included in each tree, tree depth, loss reduction, learning rate, and minimum n for split), Neural network (number of hidden layers and penalty), Naïve Bayes (smoothness and laplace), and lightGBM (number of variables included in each tree, tree depth, loss reduction and learn rate) were tuned with a Latin Hypercube search approach with internal validation on 25 bootstraps of the training data with classification performance evaluation scored as AUC. Optimal hyperparameters were used in the final models and performance was assessed on the hold-out testing data. To estimate the robustness of predictions with regards to random effects of partitioning, this workflow procedure was repeated iteratively 20 times as randomized nested cross-validation with random partitioning of samples to training and testing set at each iteration. The variability of model performances from the nested cross-validations was estimated by fitting Bayesian models and Markov Chain Monte Carlo^26^ via the tidyposterior and rstanarm packages in R, with 5000 iterations, four chains and a prior normal distribution for the random (nest) intercepts. Posterior probability distributions of mean AUC and their contrasted differences between all models were evaluated for practical equivalence. Model variable importance was assessed with permutation of variable values (iml package in R) with loss of classification error as metric, and with model specific weights when applicable (vip package in R). Molecular variables with the highest permuted importance and model specific weights were selected as candidates for analysis in the prediction cohort.

Finally, an evaluation of the literature of the molecular variables with the highest permuted importance and model specific weights was performed in PubMed by using the search terms: cytokine/gene name (both official and alternative); "Sepsis [title]"; “Prediction”. The inclusion criteria for the search were: maximum of 10 years old; species “Human”; language: English; original papers. No exclusion criteria were employed. This evaluation aimed to select molecular variables that had been both previously studied in association with sepsis and novel predictive markers of sepsis.

All candidate molecular variables with especially high importance from either univariate, multivariate in combination with literature mining approaches were selected for further analysis in the prediction cohort, as well as variables that were selected by multiple approaches.

The selected molecular variables from the screening cohort were evaluated in the prediction cohort with respect to their univariate and multivariate discrimination of sepsis and non-sepsis patients. Both the univariate and multivariate analyzes were performed according to the workflows described above. A summary of the workflow is shown in Fig. 1.

### Ethics approval and consent to participate

The study received approval from the Stockholm Regional Ethical Review Board (reference number 2016/2001–31/2 and 2018/2202). Written informed consent was obtained from all participants. This study was conducted in accordance with the Declaration of Helsinki^27^.

## Results

### Patient characteristics

A total of 551 patients with suspected infection in the ambulance and with sufficient documentation were assessed for one of two possible outcomes, sepsis (n = 230) or non-sepsis (n = 321), within the first 36 h after arrival at the ED, in accordance with the Sepsis-3 criteria^1^. Patients who did not fulfill sepsis criteria were classified as non-sepsis. Initially, a screening cohort, consisting of 46 sepsis patients and, 50 non-sepsis patients were selected chronologically from the abovementioned 551 patients, and used for evaluation and selection of candidate molecular markers. The remaining 455 patients, as a prediction cohort, were used for the final prediction modeling (patient characteristics see Table 2). There were no statistical differences of the biological sex, age, comorbidity, or in-hospital mortality between the screening and prediction cohort.

**Table 2:** Characteristics of the screening and prediction cohorts.

Variables	Screening cohort		Prediction cohort	
	Number (%) N = 96	Median (IQR)	Number (%) N = 455	Median (IQR)
Age (year)		80 (73–86)		78 (70–85)
Sex				
Male	58/96 (60.4)		273/455 (60)	
Comorbidity				
Charlson comorbidity score^28^		2 (1–4)		2 (1–4)
Outcome				
1. Sepsis	46/96 (47.9)		184/455 (40.4)	
2. Non-sepsis	50/96 (52.1)		271/455 (59.6)	
Admitted to in-hospital care	86/96 (89.6)		374/455 (82.2)	
In-hospital mortality	5/96 (5.2)		26/455 (5.7)	

*IQR* Interquartile range.

### Identification of molecular candidates for sepsis prediction in the screening cohort

**Figure 2:**
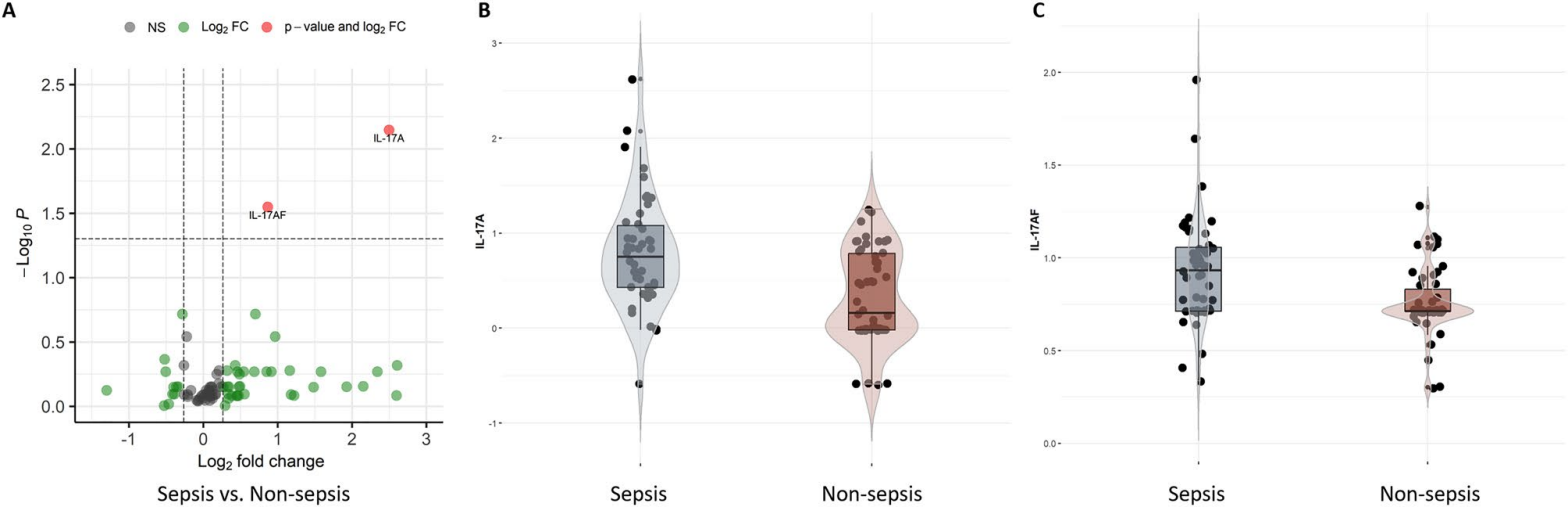
Levels of molecular markers in the screening cohort. Comparison of the levels of 74 proteins and 15 genes measured in the screening cohort. (**A**) Volcano plot of the univariate comparison between sepsis and non-sepsis patients; the threshold set to fold-change ˃ 1.2 for proteins and > 2.0 for mRNAs, and a Benjamini–Hochberg adjusted p-value ˂ 0.05. (**B**,**C**) Box-violin plots with individual values of the levels of IL-17A and IL-17AF for sepsis and non-sepsis patients, with median and interquartile range (IQR).

**Figure 3:**
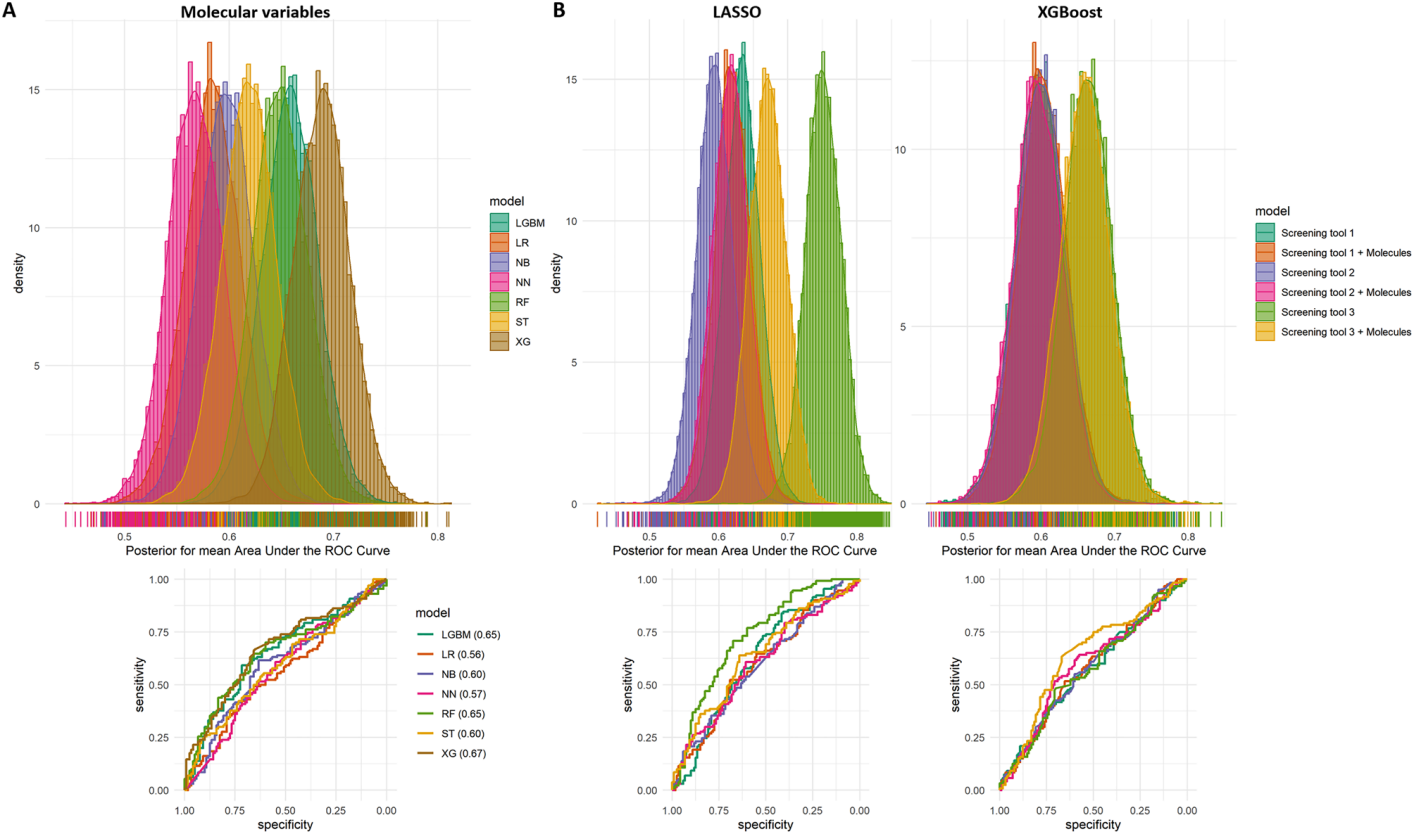
Machine learning classification of sepsis and non-sepsis patients in the screening cohort. Upper sections show the distributions of posteriors for mean area under the curve (AUC) from the nests in nested cross-validation, and lower sections show the averaged ROC curve (AUCs within parenthesis in 6A). (**A**) Distributions of posteriors for mean AUCs and averaged ROC curve from nested cross-validations with seven different algorithms trained only on proteins and gene expressions. (**B**) Distributions of posteriors for mean AUCs and averaged ROC curve from nested cross-validations trained on screening tools variables with or without added molecular variables and their interactions. *LR* Penalized regularized logistic regressions (LASSO), *RF* Random forests, *XG* XGBoosted trees, *NN* Neural network, *NB* Naïve Bayes, *LGBM* lightGBM, *ST* Stacked model of LR, XG and NN.

An inflammatory/immune panel of 74 proteins and 15 genes was analyzed in the screening cohort. Elevated levels of IL-17AF and IL-17A were observed in the septic patients group compared to the non-sepsis group in univariate comparisons (Fig. 2). In the multivariate analysis of the molecular markers, no separation between sepsis and non-sepsis patients was revealed using the unsupervised analyses with principal component analysis (PCA) (Supplementary Fig. 1A). However, with a supervised dimensional reduction approach using partial least squares (PLS), partial separation was observed (Supplementary Fig. 1B) between sepsis and non-sepsis patients, and this separation was further evaluated with supervised machine learning for sepsis prediction and molecular marker candidate selection. The results from machine learning demonstrated a moderate power of the molecular markers to separate sepsis from non-sepsis with averaged AUCs of nests between 0.57 and 0.67 for the different algorithms (Fig. 3A). The posteriors for mean AUCs showed in general a rather wide distribution for the molecule models indicating an intra-variability effect of resampling nests in the screening cohort. Evaluation of the added value of the molecular variables to the previously reported Predict Sepsis screening tools^18^ showed that in general, the molecular variables did not increase the performance of the screening tools in the screening cohort (results from Lasso regression and XGBoosted trees are shown in Fig. 3B).

**Figure 4:**
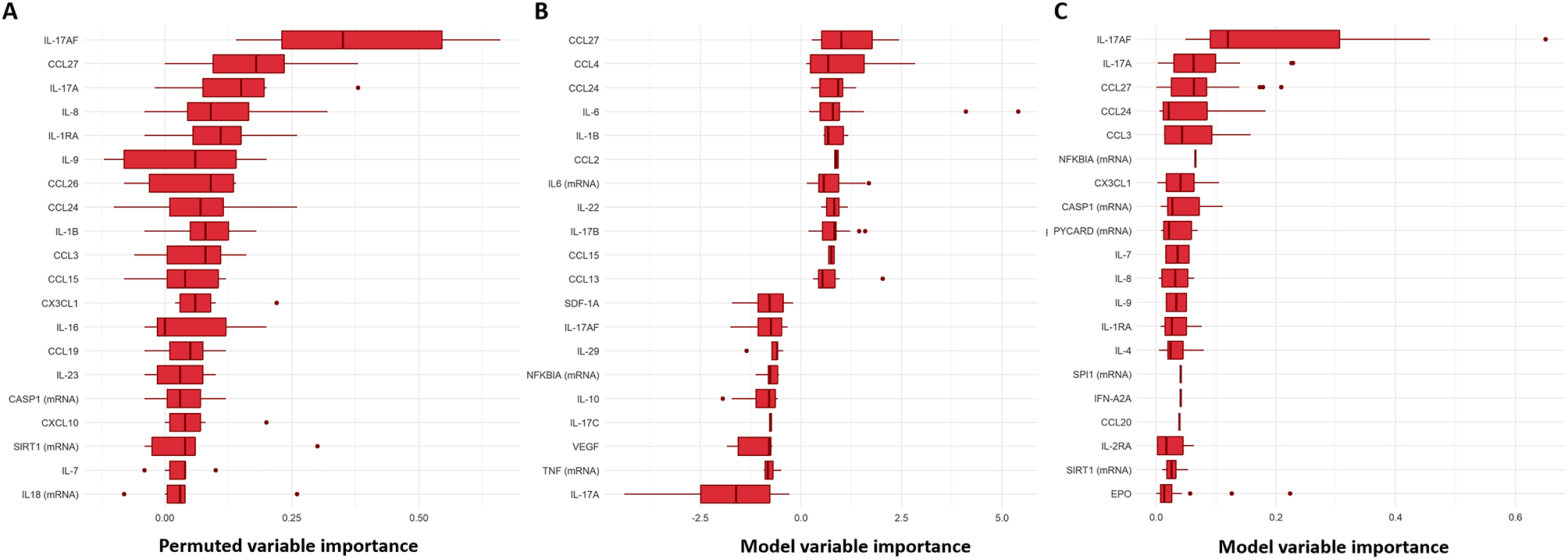
Variables with highest importance in sepsis classification models of the screening cohort. Molecules were ranked by their variable importance values from all classification models based on the molecular parameters of the screening cohort. (**A**) Model agnostic variable importance by permutation from nested cross-validations of seven different algorithms trained on all proteins and gene expressions. Model specific variable importance weights: (**B**) Coefficients for Lasso regression, and (**C**) Gini index node impurity for XGBoost. Molecules labeled with mRNA in parenthesis refers to the gene expression data. Boxplots presented with median and interquartile range (IQR).

The variable importance of the molecular variables in the nested cross-validations was evaluated by both model-agnostic importance by permutation (Fig. 4A) and by model-specific weights (Fig. 4B,C). None of these paired variable interactions showed higher importance than the original variables per se and they are therefore omitted from the figures. Thirty-three inflammatory mediators and eight genes, with higher variable importance listed in Fig. 4, together with IL-17A and IL-17AF in Fig. 2, were further considered in the selection of molecular markers candidates. The literature evaluation gave final weights to the selection of 12 molecular markers (9 proteins and 3 genes) to be evaluated in the prediction cohort; namely CCL24, CX3CL1, CCL27, CCL11, IL-17AF, IL-17A, IL-1Ra, TNF, CCL19, and genes, including *EPAS1, HIF1A*, and *NLRP3*.

### Differential expressions of the selected molecular markers between sepsis and non-sepsis patients in the prediction cohort

**Figure 5:**
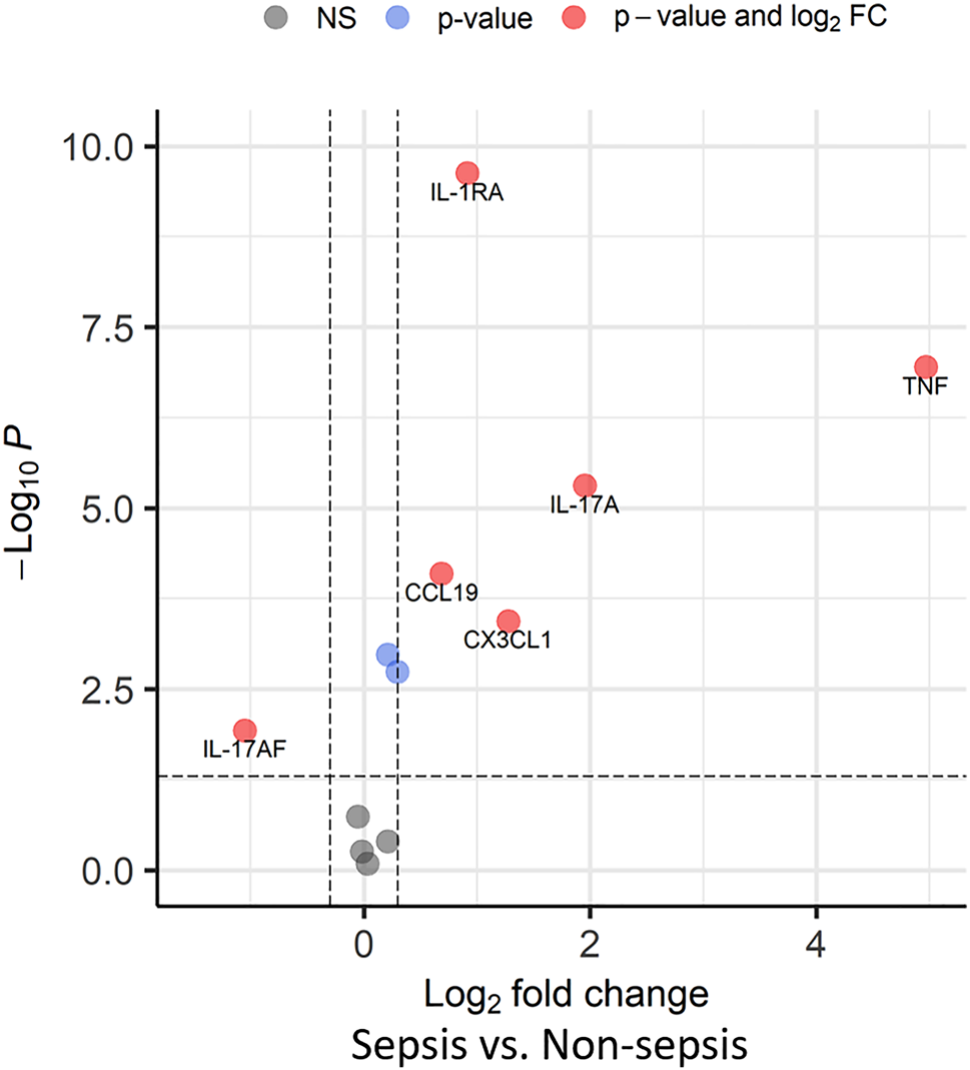
Expression levels of immune mediators and genes in the prediction cohort. Volcano plot of the univariate comparison between sepsis and non-sepsis patients. The threshold set to fold-change ˃ 1.2 for proteins and > 2.0 for mRNAs, and a Benjamini–Hochberg adjusted p-value ˂ 0.05.

Among the 12 selected molecular markers, the univariate analysis results show that levels of IL-1Ra, IL-17A, CCL19, CX3CL1, and TNF were significantly higher in plasma from the sepsis patients compared to non-sepsis patients in the prediction cohort, whereas levels of IL-17AF were higher in non-sepsis patients (Fig. 5). The multivariate analysis, similar to the screening cohort, showed that the supervised PLS, but not the unsupervised PCA, demonstrated partial separation between sepsis and non-sepsis (Supplementary Fig. 2). This separation was further evaluated for prediction of sepsis in the prediction cohort.

### Performance of sepsis prediction models with selected variables in the prediction cohort

**Figure 6:**
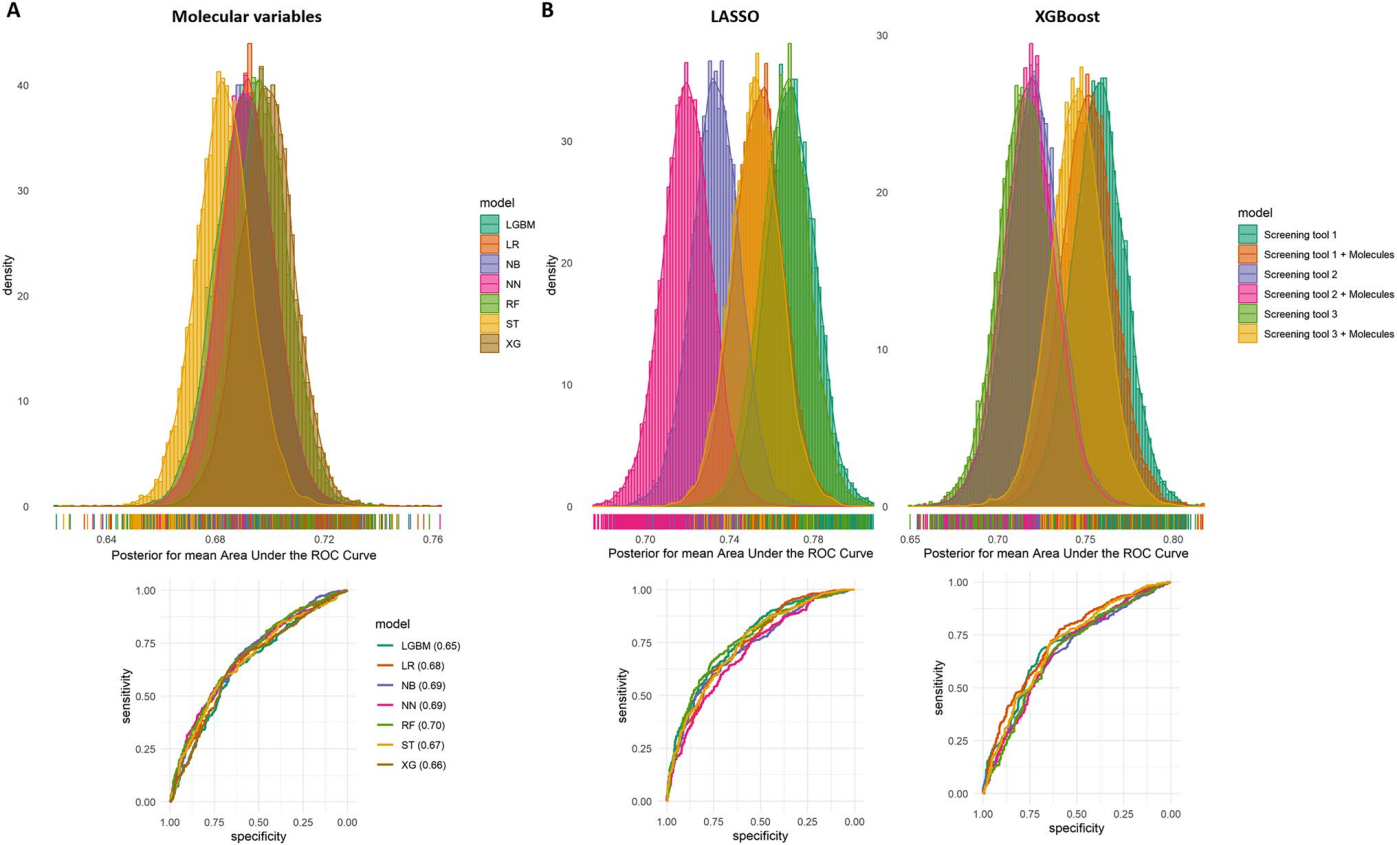
Machine learning classification of sepsis and non-sepsis patients in the prediction cohort. Upper sections show the distributions of posteriors for mean area under the curve (AUC) from the nests in nested cross-validation and lower sections show the averaged ROC curve (AUCs within parenthesis in 6A). (**A**) Distributions of posteriors for mean AUCs and averaged ROC curve from nested cross-validations with seven different algorithms trained on molecular variables alone. (**B**) Distributions of posteriors for mean AUCs and averaged ROC curve from nested cross-validations trained on screening tools variables with or without molecular variables and their interactions. *LR* Penalized regularized logistic regressions (LASSO), *RF* Random forests, *XG* XGBoosted trees, *NN* Neural network, *NB* Naïve Bayes, *LGBM* lightGBM, *ST* Stacked model of LR, XG and NN.

The evaluation of added value of the selected 12 molecular variables to the Predict Sepsis screening tools^18^ showed that the molecular variables per se did not further contribute to the predictive performance of the screening tools (Fig. 6B). Training of models on the 12 selected molecular markers based on the prediction cohort data and with the previously employed different machine learning algorithms showed moderate predictive performance with averaged AUCs between 0.65 and 0.70 (Fig. 6A). The posteriors for mean AUCs showed a smaller distribution for the molecule models in the prediction cohort compared to the screening cohort indicating less intra-variability effect of resampling nests.

**Figure 7:**
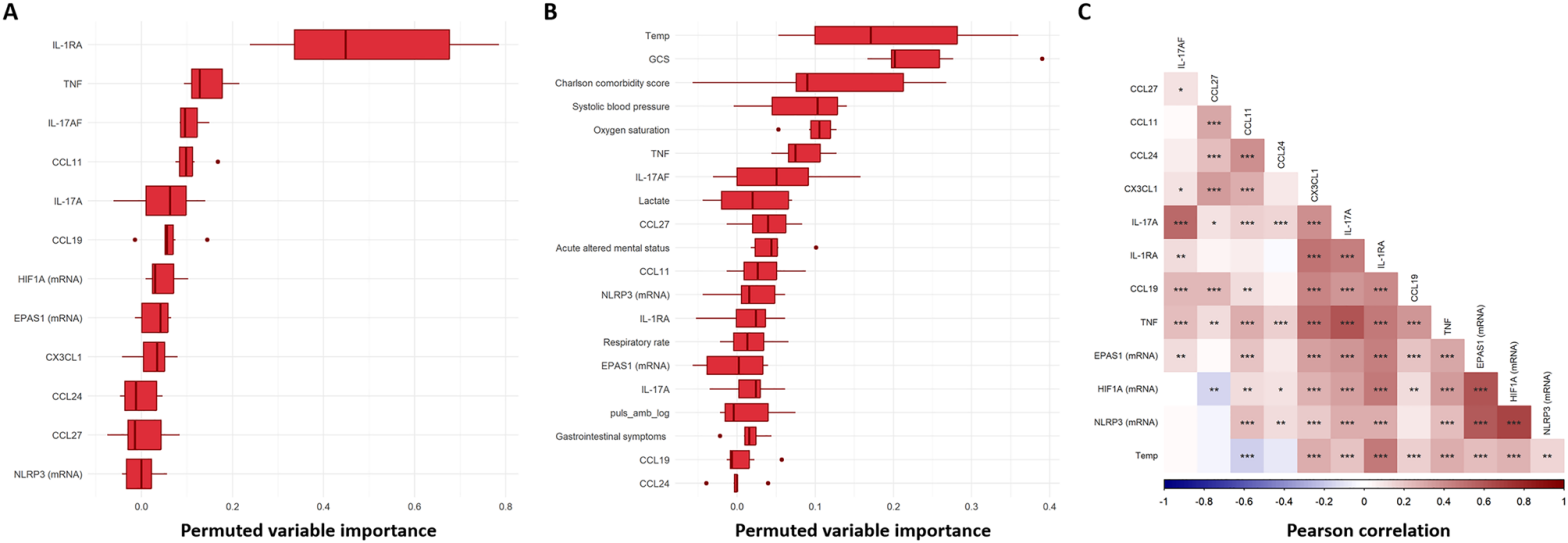
Importance of molecular and clinical variables from sepsis screening tools in the prediction cohort. (**A**) Molecules with highest model-agnostic variable importance by permutation from nested cross-validations of seven different algorithms trained on molecular markers alone. (**B**) Top-20 model-agnostic variable importance by permutation from nested cross-validations of seven different algorithms trained on all screening tools variables and molecular variables. (**C**) Pearson correlations between the molecular markers and temperature (*, ** and *** denotes a p-value ˂ 0.05, 0.01 and 0.001 respectively, color denotes correlation coefficient). Molecules labeled with mRNA in parenthesis refers to the gene expression data. Boxplots presented with median and interquartile range (IQR).

The model-agnostic variable importance, as obtained by permutation, indicates that IL-1Ra is the most important predictor among the molecular markers (Fig. 7A). Again, no variable interactions had higher importance than the original variables per se. The evaluation of permuted variable-based importance for models of the Predict Sepsis screening tools with molecular variables show higher importance for many of the clinical variables than for the selected molecular variables (Fig. 7B). A Pearson correlation matrix showed a high correlation of the screening tools variable “temperature” and several of the selected molecular variables, such as IL-1Ra (Fig. 7C), capturing much of the informative variation of the molecular markers.

### Evaluation of miss-classified subgroups of patients

**Figure 8:**
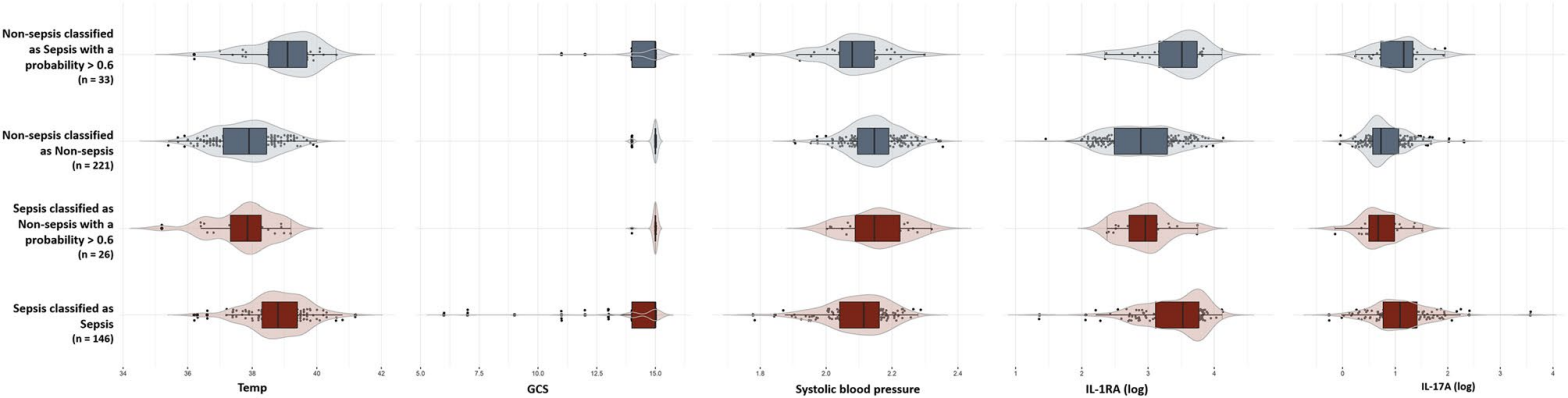
Expression of clinical and molecular parameters for miss-classified patients. Box-violin plots with individual values of the expression levels of clinical and immune parameters among miss-classified patients in all the prediction models from both sepsis and infection non-sepsis groups, with median and interquartile range (IQR). *Temp* temperature, *GSC* Glasgow Coma Scale.

To explore and understand the underlying inability to fully predict septic patients in the current cohort, an evaluation of the miss-classified patients was performed. Groups of patients who was consistently miss-classified (with probabilities above 0.6 to be classified into the other group in all nested cross-validations) was identified. As demonstrated in Fig. 8, miss-classified septic patients (n = 26) presented with milder fever, higher GCS and systolic blood pressure as well as lower IL-1Ra and IL-17A levels, while miss-classified non-sepsis patients (n = 33) demonstrated higher temperature, lower GCS, lower systolic blood pressure, and higher level of IL-1Ra and IL-17A, when comparing to the rest of their group respectively.

## Discussion

In the current study, we aimed to evaluate the additional predictive value of immunological molecular markers to the Predict Sepsis screening tools previously developed in our group^18^. The results demonstrated that when comparing the screening tools with and without added molecular markers and their interactions, this addition did not increase the predictive value. This result could be due to the high multicollinearity between clinical parameters and inflammatory mediators, which was most evident for the parameter temperature that appeared to capture most of the model informative variation of several molecular markers, such as IL-1Ra.

Molecular profiles of the host immune response reveal the key pathophysiology of sepsis and have been proposed to be better predictors of the subgroups of sepsis and outcomes^29,30^. In the current study, the prediction models based on molecular markers alone, showed a performance in terms of AUCs between 0.65 and 0.70. IL-1Ra was the most important predictor among the 12 molecular markers, followed by TNF. Additionally, the plasma levels of IL-1Ra, IL-17A, CCL19, CX3CL1, and TNF were significantly higher in septic patients compared to the non-sepsis group. Increased IL-1Ra levels have previously been observed in septic patients^31,32^, and have been suggested to be a predictor of sepsis outcome, severity^33,34^, and mortality^31^. These data were supported by the results of the currents study, where IL-Ra was found to be elevated in sepsis patients and has the highest importance value in the prediction models based on the molecular markers. TNF is another well-studied inflammatory cytokine but has not previously been associated extensively with the prediction or prognosis of sepsis. IL-17A has been found in several other studies in association to sepsis severity and mortality^35–37^ but had a moderate contribution in our prediction models. To our knowledge, neither CX3CL1 nor CCL19 have previously been evaluated as predictors in sepsis. Our data demonstrate that both these chemokines were increased in sepsis patients, with moderate predictive values among other immune mediators. IL-17AF was expressed in low levels among both septic and non-septic patients in both cohorts in our study, but IL-17AF was found to increase in the septic patients in the screening cohort compared to non-septic patients, whereas a reduced expression of this cytokine was found in sepsis patients in the prediction cohort. The discrepancy in the plasma levels of this cytokine in the patients of the screening and prediction cohort, respectively, cannot be explained but might be due to unforeseen heterogeneity among the patients.

A sub-group of sepsis patients was consistently miss-classified when building the prediction models. Interestingly, several of their clinical measurements and the expression of molecular mediators were distinct when compared to the remainder of the septic patient group. For example, miss-classified septic patients presented a milder fever, higher GSCs, and reduced IL-1Ra and IL-17A levels, which is similar to the pattern observed in the non-sepsis group of patients. This observation may illustrate a general need to stratify sepsis patients into several clinical groups or phenotypes for early diagnosis. The concept of sepsis consisting of more distinct subtypes or phenotypes, is an area of increased attention^30,38,39^. Distinct immunological phenotypes, based on transcriptomic data, have previously been associated with sepsis outcome (mortality). Increased innate immune activation or dysregulation of coagulation phenotypes was associated with higher mortality while lower mortality was associated with increased adaptive immune activation^40^. Similarly, a recent, large retrospective study identified four sepsis phenotypes from routinely available clinical data, with highly varying degree of laboratory abnormalities and dysregulated host-response markers^39^. These studies align well with our findings of consistently miss-classified patients and supports the notion that it might be more difficult to accurately predict certain sub-groups of patients. A priori knowledge about certain molecular markers and clinical variables would be a way to further inform and improve prediction models, for example by introducing stacked modeling approaches which first stratify patients, e.g. with regard to temperature, and then fit strata-specific combined clinical-molecular models, or by implementing hybrid modeling approaches with inbuilt mechanistic parts, or with distinct ratios of decisive variables, to overcome the large heterogeneity in this group of patients.

There are limitations of the study. First, the inclusion criterion was patients with suspected infection judged by the ambulance personnel based on clinical experience and presenting symptoms. This might introduce a bias that the cohort does not represent the patients that lack symptoms; a limitation due to the fact that there is no consensus criterion of infection. However, the outcomes were criteria based, including infection, a definition which has been used in the prior study^18^. Almost one third of the patients included in the current study did not have fever, supporting that the entire spectrum of sepsis was included. Second, the rather small size of the screening cohort may have limited the possibility of identifying additional molecular markers that could improve prediction performances of the models. However, the selection of candidate markers from the screening cohort was necessary, due to financing restrictions, which may have limited the selection of potentially relevant molecular candidate markers in the larger prediction cohort. While the final selection of molecular markers was primarily based on univariate and multivariate analyses in the screening cohort, the literature curation of the markers did not specifically target molecular predictors in the ambulance setting, as this is little studied, which may have introduced a minor bias. Regarding our modeling approach, we chose to establish and fit separate prediction models for the screening and for the prediction cohort, respectively, as the measured molecules were partly assessed with different molecular methods in the cohorts. However, the larger prediction cohort makes the additional assessment of variable importance in newly fitted models an attractive aim of the study and repeated nested cross-validation allowed for good estimation of model performance and evaluation of resampling influence on posterior AUC distributions. Moreover, a systematic assessment of potential interactions, between clinical-clinical, clinical-molecular, and molecular-molecular variables, was not feasible within the framework of the current study.

## Conclusions

Combing molecular markers with clinical parameters did not increase the predictive values of the screening tools, most likely due to the high multicollinearity of temperature and immune mediators. Moreover, we identified a sub-group of sepsis patients that was consistently miss-classified in our prediction models, due to their mild symptoms as well as lower expression levels of the immune mediators comparing to the remainder of the septic patients. This observation indicates that being able to initially stratify the septic patients with a priori knowledge about certain clinical and molecular parameters, could further inform and improve the prediction models for early diagnosis.

## Supplementary Information

Supplementary Figure 1.

Supplementary Figure 2.

Supplementary Legends.

Supplementary Table 1.

Supplementary Table 2.

## Data Availability

The data that used in this study are available from the corresponding author upon reasonable request.

## Abbreviations

AUC: Area under the receiver operating characteristic (ROC) curve
CASP1: Caspase 1
CCL: C–C motif chemokine ligand
CT: Cycle threshold
CV: Coefficient of variation
CX3CL: C-X3-C motif ligand
CXCL: C-X-C motif chemokine ligand
ED: Emergency department
EPAS1: Endothelial PAS domain protein 1
FC: Fold-changes
FDR: False discovery rate (FDR)
GCS: Glasgow Coma Scale
GUSB: Beta-glucuronidase
HGF: Hepatocyte growth factor
HIF1A: Hypoxia-inducible factor 1-alpha
HLA-DRA: Major histocompatibility complex, class II, DR alpha
HPRT1: Hypoxanthine–guanine phosphoribosyltransferase
HR: Heart rate
IL: Interleukin
IQR: Interquartile range
LGBM: LightGBM
LR: Penalized regularized logistic regressions (Lasso)
LOD: Limit of detection
NB: Naïve Bayes
NFKBIA: NF-kappa-B inhibitor alpha
NLRP3: NLR family pyrin domain containing 3
NN: Neural network
PCA: Principal component analysis
PLS: Partial least squares
PPIA: Peptidyl-propyl isomerase A, CyclophilinA
PYCARD: PYD and CARD domain containing
RF: Random forests
RIN: RNA integrity number
RR: Respiratory rate
SBP: Systolic blood pressure
SIRT1: Sirtuin 1
SPI1: Spi-1 proto-oncogene
ST: Stacked model of LR, XG and NN
Temp: Temperature
TGF-α: Transforming growth factor-alpha
TNF: Tumor necrosis factor
UBC: Ubiquitin C
XG: XGBoosted trees

## References

[1] Singer, M. The new sepsis consensus definitions (Sepsis-3): The good, the not-so-bad, and the actually-quite-pretty. *Intensive Care Med.* **42**, 2027–2029 (2016). 27815587 10.1007/s00134-016-4600-4

[2] Bauer, M. *et al.* Mortality in sepsis and septic shock in Europe, North America and Australia between 2009 and 2019—Results from a systematic review and meta-analysis. *Crit. Care.* **24**, 239 (2020). 32430052 10.1186/s13054-020-02950-2 PMC7236499

[3] Mouncey, P. R. *et al.* Trial of early, goal-directed resuscitation for septic shock. *N. Engl. J. Med.* **372**, 1301–1311 (2015). 25776532 10.1056/NEJMoa1500896

[4] Mellhammar, L. *et al.* Sepsis incidence: A population-based study. *Open Forum Infect. Dis.* **3**, 207 (2016). 10.1093/ofid/ofw207 PMC5144652 27942538

[5] Ljungstrom, L., Andersson, R. & Jacobsson, G. Incidences of community onset severe sepsis, Sepsis-3 sepsis, and bacteremia in Sweden—A prospective population-based study. *PLoS ONE* **14**, e0225700 (2019). 31805110 10.1371/journal.pone.0225700 PMC6894792

[6] Rudd, K. E. *et al.* Global, regional, and national sepsis incidence and mortality, 1990–2017: Analysis for the Global Burden of Disease Study. *Lancet* **395**, 200–211 (2020). 31954465 10.1016/S0140-6736(19)32989-7 PMC6970225

[7] World Health Organization 2023. World Health Organization website, Sepsis. https://www.who.int/news-room/fact-sheets/detail/sepsis. Accessed 29 March 2023.

[8] Seymour, C. W. *et al.* Time to treatment and mortality during mandated emergency care for sepsis. *N. Engl. J. Med.* **376**, 2235–2244 (2017). 28528569 10.1056/NEJMoa1703058 PMC5538258

[9] Asner, S. A., Desgranges, F., Schrijver, I. T. & Calandra, T. Impact of the timeliness of antibiotic therapy on the outcome of patients with sepsis and septic shock. *J. Infect.* **82**, 125–134 (2021). 33722641 10.1016/j.jinf.2021.03.003

[10] Im, Y. *et al.* Time-to-antibiotics and clinical outcomes in patients with sepsis and septic shock: A prospective nationwide multicenter cohort study. *Crit. Care.* **26**, 19 (2022). 35027073 10.1186/s13054-021-03883-0 PMC8756674

[11] Axelsson, C. *et al.* The early chain of care in patients with bacteraemia with the emphasis on the prehospital setting. *Prehosp. Disaster Med.* **31**, 272–277 (2016). 27026077 10.1017/S1049023X16000339

[12] Wang, H. E., Weaver, M. D., Shapiro, N. I. & Yealy, D. M. Opportunities for Emergency Medical Services care of sepsis. *Resuscitation* **81**, 193–197 (2010). 20006419 10.1016/j.resuscitation.2009.11.008 PMC4028958

[13] Studnek, J. R., Artho, M. R., Garner, C. L. Jr. & Jones, A. E. The impact of emergency medical services on the ED care of severe sepsis. *Am. J. Emerg. Med.* **30**, 51–56 (2012). 21030181 10.1016/j.ajem.2010.09.015 PMC3032016

[14] Smyth, M. A., Brace-McDonnell, S. J. & Perkins, G. D. Identification of adults with sepsis in the prehospital environment: A systematic review. *BMJ Open* **6**, e011218 (2016). 27496231 10.1136/bmjopen-2016-011218 PMC4985978

[15] Wallgren, U. M., Castrén, M., Svensson, A. E. & Kurland, L. Identification of adult septic patients in the prehospital setting: A comparison of two screening tools and clinical judgment. *Eur. J. Emerg. Med.* **21**, 260–265 (2014). 24080997 10.1097/MEJ.0000000000000084

[16] Wallgren, U. M., Antonsson, V. E., Castrén, M. K. & Kurland, L. Longer time to antibiotics and higher mortality among septic patients with non-specific presentations—A cross sectional study of Emergency Department patients indicating that a screening tool may improve identification. *Scand. J. Trauma Resusc. Emerg. Med.* **24**, 1 (2016). 26733395 10.1186/s13049-015-0193-0 PMC4702378

[17] Alam, N. *et al.* Exploring the performance of the National Early Warning Score (NEWS) in a European emergency department. *Resuscitation* **90**, 111–115 (2015). 25748878 10.1016/j.resuscitation.2015.02.011

[18] Wallgren, U. M., Sjolin, J., Jarnbert-Pettersson, H. & Kurland, L. The predictive value of variables measurable in the ambulance and the development of the Predict Sepsis screening tools: A prospective cohort study. *Scand. J. Trauma Resusc. Emerg. Med.* **28**, 59 (2020). 32586337 10.1186/s13049-020-00745-6 PMC7318751

[19] Suffoletto, B. *et al.* Prediction of serious infection during prehospital emergency care. *Prehosp. Emerg. Care.* **15**, 325–330 (2011). 21524204 10.3109/10903127.2011.561411

[20] Hotchkiss, R. S., Monneret, G. & Payen, D. Immunosuppression in sepsis: A novel understanding of the disorder and a new therapeutic approach. *Lancet Infect. Dis.* **13**, 260–268 (2013). 23427891 10.1016/S1473-3099(13)70001-X PMC3798159

[21] Hung, S. K., Lan, H. M., Han, S. T., Wu, C. C. & Chen, K. F. Current evidence and limitation of biomarkers for detecting sepsis and systemic infection. *Biomedicines.* **8**, 494 (2020). 33198109 10.3390/biomedicines8110494 PMC7697922

[22] Pierrakos, C. & Vincent, J. L. Sepsis biomarkers: A review. *Crit. Care.* **14**, R15 (2010). 20144219 10.1186/cc8872 PMC2875530

[23] Pierrakos, C., Velissaris, D., Bisdorff, M., Marshall, J. C. & Vincent, J. L. Biomarkers of sepsis: Time for a reappraisal. *Crit. Care.* **24**, 287 (2020). 32503670 10.1186/s13054-020-02993-5 PMC7273821

[24] Barichello, T., Generoso, J. S., Singer, M. & Dal-Pizzol, F. Biomarkers for sepsis: More than just fever and leukocytosis—A narrative review. *Crit. Care.* **26**, 14 (2022). 34991675 10.1186/s13054-021-03862-5 PMC8740483

[25] Laurell, H. *et al.* Correction of RT-qPCR data for genomic DNA-derived signals with ValidPrime. *Nucleic Acids Res.* **40**, e51 (2012). 22228834 10.1093/nar/gkr1259 PMC3326333

[26] Carlin, B. P. & Chib, S. Bayesian model choice via Markov chain Monte Carlo methods. *J. R. Stat. Soc. Ser. B (Methodol.)* **57**, 473–484 (1995). 10.1111/j.2517-6161.1995.tb02042.x

[27] World Medical Association Declaration of Helsinki. ethical principles for medical research involving human subjects. *J. Am. Coll. Dent.* **81**, 14–18 (2014). 25951678

[28] Charlson, M. E., Pompei, P., Ales, K. L. & MacKenzie, C. R. A new method of classifying prognostic comorbidity in longitudinal studies: Development and validation. *J. Chronic Dis.* **40**(5), 373–383 (1987). 3558716 10.1016/0021-9681(87)90171-8

[29] Davenport, E. E. *et al.* Genomic landscape of the individual host response and outcomes in sepsis: A prospective cohort study. *Lancet Respir. Med.* **4**, 259–271 (2016). 26917434 10.1016/S2213-2600(16)00046-1 PMC4820667

[30] Sweeney, T. E. *et al.* A community approach to mortality prediction in sepsis via gene expression analysis. *Nat. Commun.* **9**, 694 (2018). 29449546 10.1038/s41467-018-03078-2 PMC5814463

[31] Potjo, M. *et al.* Interleukin-10 and interleukin-1 receptor antagonist distinguish between patients with sepsis and the systemic inflammatory response syndrome (SIRS). *Cytokine* **120**, 227–233 (2019). 31125901 10.1016/j.cyto.2019.05.015

[32] Chen, P., Stanojcic, M. & Jeschke, M. G. Septic predictor index: A novel platform to identify thermally injured patients susceptible to sepsis. *Surgery.* **163**, 409–414 (2018). 29129362 10.1016/j.surg.2017.08.010 PMC5939595

[33] Jekarl, D. W. *et al.* Diagnosis and prognosis of sepsis based on use of cytokines, chemokines, and growth factors. *Dis. Mark.* **2019**, 1089107 (2019). 10.1155/2019/1089107 PMC6754872 31583025

[34] Taneja, I. *et al.* Combining biomarkers with EMR data to identify patients in different phases of sepsis. *Sci. Rep.* **7**, 10800 (2017). 28883645 10.1038/s41598-017-09766-1 PMC5589821

[35] Liu, Y., Wang, X. & Yu, L. Th17, rather than Th1 cell proportion, is closely correlated with elevated disease severity, higher inflammation level, and worse prognosis in sepsis patients. *J. Clin. Lab. Anal.* **35**, e23753 (2021). 33704828 10.1002/jcla.23753 PMC8128311

[36] Li, G., Zhang, L., Han, N., Zhang, K. & Li, H. Increased Th17 and Th22 cell percentages predict acute lung injury in patients with sepsis. *Lung* **198**, 687–693 (2020). 32462370 10.1007/s00408-020-00362-1

[37] Costa, R. T. *et al.* T helper type cytokines in sepsis: Time-shared variance and correlation with organ dysfunction and hospital mortality. *Braz. J. Infect. Dis.* **23**, 79–85 (2019). 31112675 10.1016/j.bjid.2019.04.008 PMC9425672

[38] Yende, S. *et al.* Long-term host immune response trajectories among hospitalized patients with sepsis. *JAMA Netw. Open.* **2**, e198686 (2019). 31390038 10.1001/jamanetworkopen.2019.8686 PMC6686981

[39] Seymour, C. W. *et al.* Derivation, validation, and potential treatment implications of novel clinical phenotypes for sepsis. *JAMA* **321**, 2003–2017 (2019). 31104070 10.1001/jama.2019.5791 PMC6537818

[40] Sweeney, T. E. *et al.* Unsupervised analysis of transcriptomics in bacterial sepsis across multiple datasets reveals three robust clusters. *Crit. Care Med.* **46**, 915–925 (2018). 29537985 10.1097/CCM.0000000000003084 PMC5953807

