# Author Correction: Predicting sepsis using a combination of clinical information and molecular immune markers sampled in the ambulance

**DOI:** 10.1038/s41598-024-72325-y

**Published:** 2024-09-12

**Authors:** Kedeye Tuerxun, Daniel Eklund, Ulrika Wallgren, Katharina Dannenberg, Dirk Repsilber, Robert Kruse, Eva Särndahl, Lisa Kurland

**Affiliations:** 1 School of Medical Sciences, Faculty of Medicine and Health, Örebro University, Örebro, Sweden; 2 Inflammatory Response and Infection Susceptibility Centre, (iRiSC), Faculty of Medicine and Health, Örebro University, Örebro, Sweden; 3 Gustavsbergs Vårdcentral, Gustavsberg, Stockholm, Sweden; 4 Department of Clinical Research Laboratory, Faculty of Medicine and Health, Örebro University, Örebro, Sweden; 5 Department of Emergency Medicine, Örebro University Hospital, Örebro, Sweden

Correction to: *Scientific Reports* 10.1038/s41598-023-42081-6, published online 10 September 2023

The original version of this Article contained an error in Figure 1, in which the numbers of the Sepsis and Infection non-sepsis patients in the Prediction Cohort were not correct. The original Figure 1 and accompanying legend appear below.

**Fig. 1:**
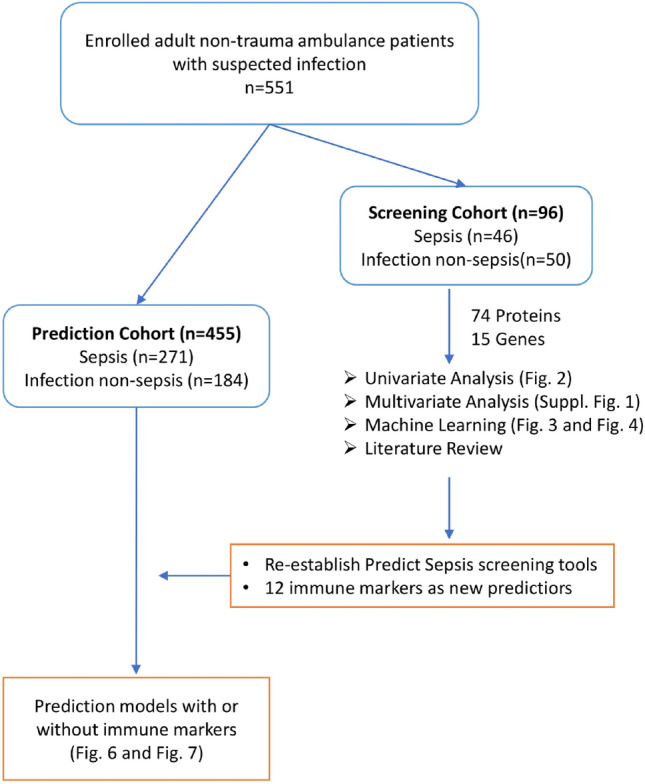
Schematic illustration of the outline of current study.

