# Supplementary figures and images for: Predicting sepsis using a combination of clinical information and molecular immune markers sampled in the ambulance
Source: Sci Rep. 2023 Sep 10;13:14917. doi: 10.1038/s41598-023-42081-6 (PMC10493220; doi:10.1038/s41598-023-42081-6)

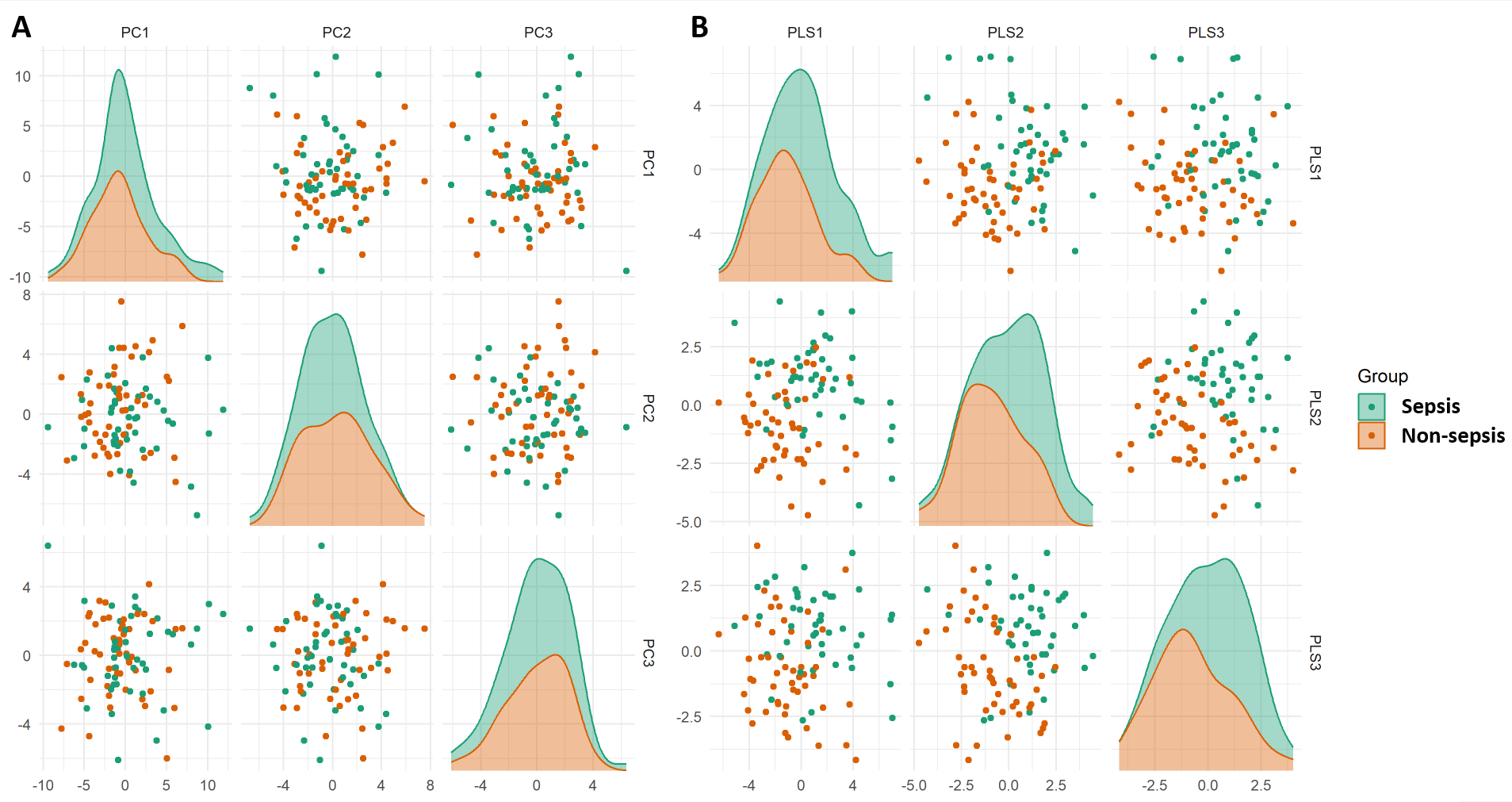

Supplement: Supplementary file 1 — Supplementary Figure 1. [file 41598_2023_42081_MOESM1_ESM.png]

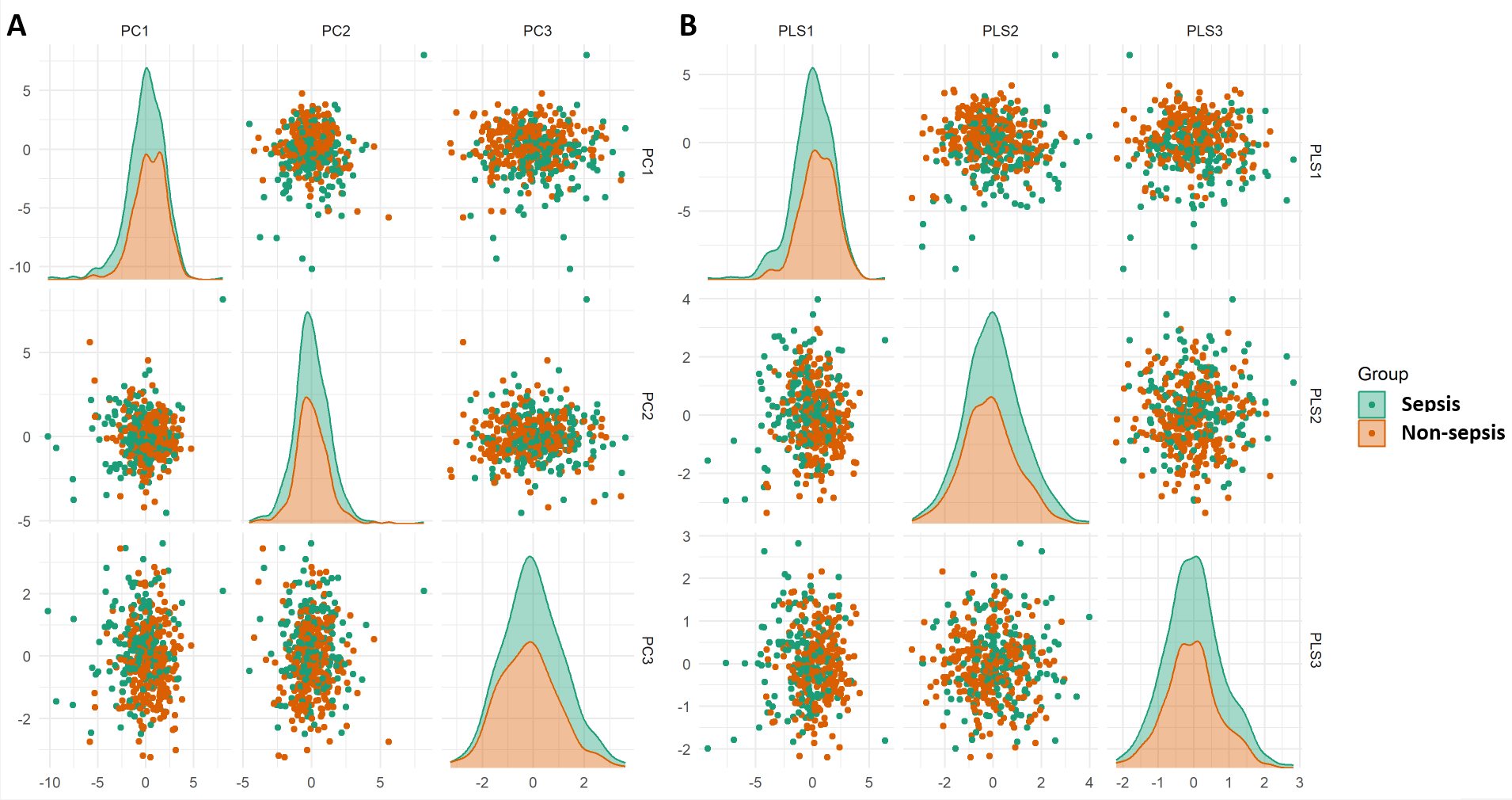

Supplement: Supplementary file 2 — Supplementary Figure 2. [file 41598_2023_42081_MOESM2_ESM.png]
