# Supplementary material for: Predicting sepsis using a combination of clinical information and molecular immune markers sampled in the ambulance
Source: Sci Rep. 2023 Sep 10;13:14917. doi: 10.1038/s41598-023-42081-6 (PMC10493220; doi:10.1038/s41598-023-42081-6)
Supplement: Supplementary file 3 — Supplementary Legends. [file 41598_2023_42081_MOESM3_ESM.docx]

**Supplemental figure 1. Distribution of variation in molecular markers in the screening cohort.** Unsupervised dimensionality reduction of expressions with A) Principal Component Analysis (PCA), and supervised dimensionality reduction with B) Partial Least Squares (PLS) for separation of sepsis and non-sepsis patients. Figures are overlaid with color representing sepsis and non-sepsis patients.

**Supplemental figure 2. Distribution of variation of immune makers in the prediction** **cohort.** Distribution of variation in immune mediator and gene expressions with respect to sepsis and non-sepsis using unsupervised dimensionality reduction of expressions with A) Principal Component Analysis (PCA), and supervised dimensionality reduction with B) Partial Least Squares (PLS) for separation of sepsis and non-sepsis patients. Figures are overlaid with color representing sepsis and non-sepsis patients.
