# Supplementary material for: Predicting sepsis using a combination of clinical information and molecular immune markers sampled in the ambulance
Source: Sci Rep. 2023 Sep 10;13:14917. doi: 10.1038/s41598-023-42081-6 (PMC10493220; doi:10.1038/s41598-023-42081-6)
Supplement: Supplementary file 4 — Supplementary Table 1. [file 41598_2023_42081_MOESM4_ESM.docx]

**Supplemental table 1. Detection limits of analyzed proteins and average coefficient of variation (CV) in the prediction** **cohort.**

| **Protein** | **LOD (pg/mL)** | **Avg. CV%** |
| --- | --- | --- |
| CCL24 | 3.1 - 6000 | 7.8 |
| CX3CL1 | 102 - 181000 | 5.7 |
| CCL27 | 1.8 - 4200 | 5.6 |
| CCL11 | 3.2 - 4800 | 10.6 |
| IL-17AF | 1.8 - 18400 | 9.6 |
| IL-17A | 2.1 - 23400 | 10.4 |
| IL-1Ra | 1.7 - 5000 | 4.8 |
| TNF | 0.51 - 3700 | 11.5 |
| CCL19 | 0.67 -2000 | 6.4 |

LOD: Limit of Detection. Avg.: Average. CV: Coefficient of Variation. CCL: C-C Motif Chemokine Ligand. CX3CL: C-X3-C motif Ligand. IL: Interleukin. TNF: Tumor Necrosis Factor.
