# Supplementary material for: Predicting sepsis using a combination of clinical information and molecular immune markers sampled in the ambulance
Source: Sci Rep. 2023 Sep 10;13:14917. doi: 10.1038/s41598-023-42081-6 (PMC10493220; doi:10.1038/s41598-023-42081-6)
Supplement: Supplementary file 5 — Supplementary Table 2. [file 41598_2023_42081_MOESM5_ESM.docx]

**Supplemental table 2. Description of assays in real-time PCR in the screening cohort.**

| **Gene** | **TaqMan assay ID** | **Assay** |
| --- | --- | --- |
| *HPRT1* | Hs02800695_m1 | Reference Gene |
| *NLRP3* | Hs00918082_m1 | Target Gene |
| *PYCARD* | Hs01547324_m1 | Target Gene |
| *CASP1* | Hs00354836_m1 | Target Gene |
| *IL1B* | Hs01555410_m1 | Target Gene |
| *IL18* | Hs01038788_m1 | Target Gene |
| *IL6* | Hs00174131_m1 | Target Gene |
| *TNF* | Hs00174128_m1 | Target Gene |
| *IL10* | Hs00961622_m1 | Target Gene |
| *IL1RN* | Hs00893626_m1 | Target Gene |
| *HIF1A* | Hs00153153_m1 | Target Gene |
| *HLA-DRA* | Hs00219575_m1 | Target Gene |
| *SPI1* | Hs02786711_m1 | Target Gene |
| *NFKBIA* | Hs00355671_g1 | Target Gene |
| *SIRT1* | Hs01009006_m1 | Target Gene |
| *EPAS1* | Hs01026149_m1 | Target Gene |

*HPRT1*: Hypoxanthine-guanine phosphoribosyltransferase. *NLRP3*: NLR family pyrin domain containing 3. *PYCARD*: PYD And CARD Domain Containing. *CASP1*: Caspase 1. *IL*: Interleukin. *TNF*: Tumor Necrosis Factor. *HIF1A*: Hypoxia-inducible factor 1-alpha. *HLA-DRA*: Major histocompatibility complex, class II, DR alpha. *SPI1*: Spi-1 Proto-Oncogene. *NFKBIA*: NF-Kappa-B Inhibitor Alpha. *EPAS1*: Endothelial PAS Domain Protein 1. *SIRT1*: Sirtuin 1.
